# A Comparative Study of Platysmal Myoneurectomy With Variations of Selective Neurectomies in Post‐Facial Palsy Synkinesis

**DOI:** 10.1002/micr.70040

**Published:** 2025-02-27

**Authors:** Maria Paranhos Barbot, Catriona Neville, Tamsin Gwynn, Karen Young, Crystal Selley‐West, Raman Malhotra, Charles Nduka, Ruben Yap Kannan

**Affiliations:** ^1^ Brighton & Sussex Medical School Brighton UK; ^2^ Department of Plastic Surgery Queen Victoria Hospital East Grinstead UK

**Keywords:** facial palsy, platysma myoneurectomy, selective neurectomy

## Abstract

Synkinesis is a distressing sequela of facial palsy. The main treatment for this condition has been Botulinum toxin injections. However, more recently, selective neurolysis/neurectomies have shown promise, with some variations in practice evolving over time. A clinical review of practice was performed on sixty‐eight patients (*n* = 68) with Bell's palsy over five years (2018–2023) who underwent synkinesis surgery, following facial palsy. Comparisons were made between platysmal myoneurectomy only (Group I), platysmal myoneurectomy and zygomaticus major/levator labii superior direct neurotization (Group II), platysmal myoneurectomy and depressor anguli oris (DAO) selective neurectomy (Group III) and platysmal myoneurectomy + DAO and buccinator selective neurectomies (Group IV). The following outcome measures were used: Sunnybrook Facial Grading Scale (FGS), Facial Clinimetric Evaluation (FaCE), Facial Disability Index (FDI), and Synkinesis Assessment Questionnaire (SAQ). Statistical analyses were performed using the student's *t*‐test and ANOVA. There was an overall improvement for patients pre‐ and post‐operatively (42.34 ± 16.37 vs. 65.12 ± 14.34; Student's *t*‐test, *p* < 0.0001). All FGS scores showed statistically significant (*p* < 0.05) improvement. FDI‐physical, SAQ, and FaCE showed statistically significant improvements, apart from the FDI‐social (*p* = 0.08). Qualitatively, all procedures showed significant improvement compared with their pre‐op state, with Group IV showing the best results among platysmal myoneurectomy variants in terms of repose, volitional movements, and synkinesis reduction compared with its contemporaries.

## 1 Introduction

Facial synkinesis is a distressing long‐term complication of facial palsy (Shokri et al. 2021). Synkinesis refers to involuntary facial muscle contractions during voluntary movement, which cause severe functional limitations by affecting facial expression, speaking, eating, and drinking (Shokri et al. 2021). These limitations are linked to patient depression and anxiety, causing significant functional, psychological, and social morbidity (Walker et al. 2012).

Selective neurectomy involves the resection of facial nerve branches responsible for synkinesis activity (Shokri et al. 2021). This reduces synkinesis by releasing tight agonistic muscles and reducing neural input to antagonistic muscles (Shokri et al. 2021). The nerves are selected using botulinum toxin A (BTX‐A) injections that identify the areas of nerve supply that benefited the most from them to create a customized BTX‐A map (Sadiq et al. 2012). The Sunnybrook Facial Grading Score (FGS) which monitors physical improvement, aids the creation of these BTX‐A maps, thereby providing a prognostic model of the longer‐term impacts of selective neurectomy in the respective areas that were injected (Neely et al. 2010; van Veen et al. 2018). This selection technique has resulted in a plethora of surgical techniques, but there has not been evidence showing which surgical variation is most effective.

This study aims to (i) evaluate the effectiveness of platysmal myoneurectomies in reducing synkinesis in facial palsy patients, (ii) objectively assess which variation of selective neurectomies, in combination with platysmal myoneurectomy, produces the most significant improvement in post‐facial palsy synkinesis, and (iii) if the BTX‐A mapping is a good selection technique for the nerves that should undergo selective neurectomy to improve synkinesis.

## 2 Materials and Methods

An audit of clinical practice on sixty‐eight patients (*n* = 68) with post‐facial palsy synkinesis over five years (2018–2023) was conducted on patients who underwent plastysmal myoneurectomies at a national referral center for facial palsy: the Queen Victoria Hospital (QVH), East Grinstead, UK. Two plastic surgeons specializing in facial palsy surgery performed the surgery supported by a specialist facial therapy team. The participants were from all parts of the United Kingdom with no specific gender bias and were over 18 years old.

At QVH, the East Grinstead Grades of Stiffness (EGGS) guide the management of facial palsy sequelae. It is a descriptive scale that divides patients into three categories, providing a stepwise approach to management (Neville et al. 2022). At QVH, 45% of patients responded to facial rehabilitation alone, while 50% required both facial rehabilitation and chemodenervation (Neville et al. 2022). About 5% of patients receiving facial therapy and chemodenervation agreed to have surgery due to their neck tightness symptoms (Neville et al. 2022). Chemodenervation was achieved using BTX‐A injections in all cases.

**FIGURE 1:**
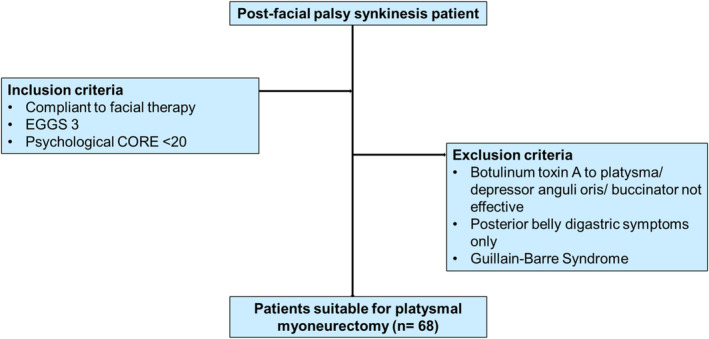
Flowchart of inclusion and exclusion criteria for undergoing surgery. Assesses the eligibility criteria for facial palsy patients to undergo platysmal myoneurectomy surgery.

The eligibility criteria for this surgery are summarized in Figure 1. Patients had their psychological CORE scores calculated by the psychological therapists to assess the impact of facial palsy on mental health, where those with high CORE scores were deemed psychologically vulnerable (Barkham et al. 2005; Pasquale et al. 2021). Patients were referred to psychological therapists to improve their mental health and were reassessed before surgery as appropriate (Barkham et al. 2005; Pasquale et al. 2021). Screening patients pre‐operatively ensured that patients were psychologically prepared for surgery and aimed to maximize compliance with the surgical process, as without addressing these underlying psychological issues, surgery could have proven to be counterproductive for these patients (Bradbury et al. 2006). Another vital aspect of surgical management is facial therapy, so patients must comply with it. Patients were excluded from surgery if they were not responsive to BTX‐A injections to the platysma, depressor anguli oris (DAO), or buccinator muscles. The customized BTX‐A maps for each patient‐guided surgery by highlighting the most affected anatomical landmarks by the muscle tightening with facial expressions. It is important to note that patients with severe contractures of the muscles may not have a clear improvement in response to BTX. This is a limitation, and clinical correlation was advised in these instances. Otherwise, the lack of response to chemodenervation predicts that surgery will not be effective (Dall'Angelo et al. 2014). If patients were only experiencing posterior belly digastric (PBG) muscle symptoms, they should not be included in this cohort, as the surgery does not alleviate these symptoms. This muscle is vital for swallowing, and its involvement in surgery could cause detrimental complications (Kim and Loukas 2019). Patients with PBG synkinesis are best treated with chemodenervation (Pescarini et al. 2021). Also, patients with Guillain–Barre syndrome did not participate because our early experience showed that this cohort did not respond well to this surgery.

Subsequently, the patients who fulfilled the eligibility criteria had a BTX‐A map created for them. According to this, patients were divided into four groups to determine which nerves would undergo selective neurectomy. Group I was used to describe the patients that underwent platysmal myoneurectomy only (*n* = 21), Group II for platysmal myoneurectomy and cervical branch direct neurotization of zygomaticus major (ZM)/levator labii superioris (LLS) (*n* = 12), Group III for platysmal myoneurectomy and DAO selective neurectomy (*n* = 27), and Group IV for platysmal myoneurectomy and DAO & buccinator selective neurectomy (*n* = 8). All groups have a platysmal myoneurectomy; the difference between each group is the addition of the procedures described. Incorporating selective neurectomies aimed to alter vectors to downgrade the neural input to a combination of muscles that affect the different surgical groups.

All patients had a retro‐tragal facelift access incision followed by a deep plane facelift as described by Azizzadeh et al. (Azizzadeh and Frisenda 2018), after which the cervical branch from the facial nerve was identified at the M‐point (Kannan 2024). For platysmal myoneurectomies, the platysma from the M‐point to the upper border of the thyroid cartilage was transected to minimize risk to the marginal mandibular nerve. Posterior and superior to the M‐point, the platysma was dissected up to the lower border of the mandible as it fused with the superficial muscular aponeurotic system (SMAS). This was the baseline procedure across all four groups. In Group II, the cervical branch was identified and transected, and a reverse sural nerve graft was coapted to the cervical branch and directly implanted into the ipsilateral ZM muscle as per the direct muscle neurotization technique described by Terzis et al. (Terzis and Karypidis 2011). In Group III, once the platysma was resected and the SMAS dissected, the facial nerve was followed to its terminal branches, and using the nerve stimulator (MedTronic NIM 3.0, USA) the superior branch to the DAO muscle was transected, thereby down‐signaling DAO innervation. Group IV's surgery was performed by doing Group III's surgery and adding the transection to the inferolateral muscular branch of the buccinator muscle to reduce neural input in this vector.

Assessment of physical and psychological outcomes was measured using clinician‐reported and patient self‐reported tools to ensure a holistic evaluation of this surgery. The FGS is a clinician‐graded tool that focuses on the patient's physical outcomes and is a widely used assessment tool for post‐facial palsy synkinesis (Neely et al. 2010). It is a categorical scale that systematically evaluates specific areas of the face to assess repose, voluntary movement, and synkinesis (Neely et al. 2010). Different clinicians calculated patients' FGS scores, which could cause random errors resulting in possible confounding. However, a QVH multidisciplinary facial palsy team audit found good inter‐ and intra‐rater reliability, reducing this risk (Neville et al. 2022). Furthermore, all assessments were done in real time, reducing selection bias as the evaluators, at the time, did not know this was for a study. The patient self‐reported outcomes were the Synkinesis Assessment Questionnaire (SAQ), Facial Clinimetric Evaluation (FaCE), and the Facial Disability Index (FDI) (Mehta et al. 2007; Kahn et al. 2001; Van Swearingen and Brach 1996). These tools assessed both physical and psychological aspects of facial nerve dysfunction by using a questionnaire that produces reliable and valid results (Mehta et al. 2007; Kahn et al. 2001; Van Swearingen and Brach 1996). For analysis, the FDI was split up and assessed as FDI‐physical and FDI‐social to pinpoint changes in the physical and social patient outcomes (Van Swearingen and Brach 1996).

Data was collected using the assessment tools pre‐ and post‐operatively. The postoperative FGS and self‐reported outcomes were measured after the patient recovered and engaged in facial therapy sessions at their three‐month appointment. All patients resumed facial therapy at least four weeks after the surgery, reducing possible confounding factors. Involvement with this significantly improves functional recovery and reduces the number of chemodenervations the patient will need (Lindsay et al. 2010).

The data collection was from the hospital digital record system called EVOLVE, which has patient information and completed assessment tools. The data was anonymized and added to Microsoft Excel to calculate averages and perform statistical analyses.

A student *t*‐test analyzed the pre‐ and post‐operative data and the improvement of each FGS parameter between the four surgical groups. The two‐way analysis of variance (ANOVA) assessed the pre‐ and post‐operative data between the groups and determined the surgery and group effect on the results. The baseline FGS parameters for each surgical group could differ, so a two‐way ANOVA was calculated to assess this potential confounder. These statistical analyses determine if there is a more beneficial surgery or if BTX‐A mapping was an effective selection technique in determining the appropriate surgery variation for each patient. The statistical significance for the data analysis is *p* < 0.05.

The STROBE (Strengthening The Reporting of Observational Studies in Epidemiology) checklists and guidelines were used to strengthen the study design. The study conforms to the Helsinki guidelines on ethics. This study was registered with and approved by the QVH Audit and Research Department before being conducted (Audit number: 418).

## 3 Results

The average age of the sixty‐eight participants (*n* = 68) in this clinical practice review was 53 years old, and the female‐to‐male ratio is 15:2 (60 females and 8 males). Facial palsy has an equal incidence in both sexes, but in this cohort, there was greater female participation implying women could be more sensitive to this condition and its sequelae, and therefore seek more treatment (Neville et al. 2022).

**FIGURE 2:**
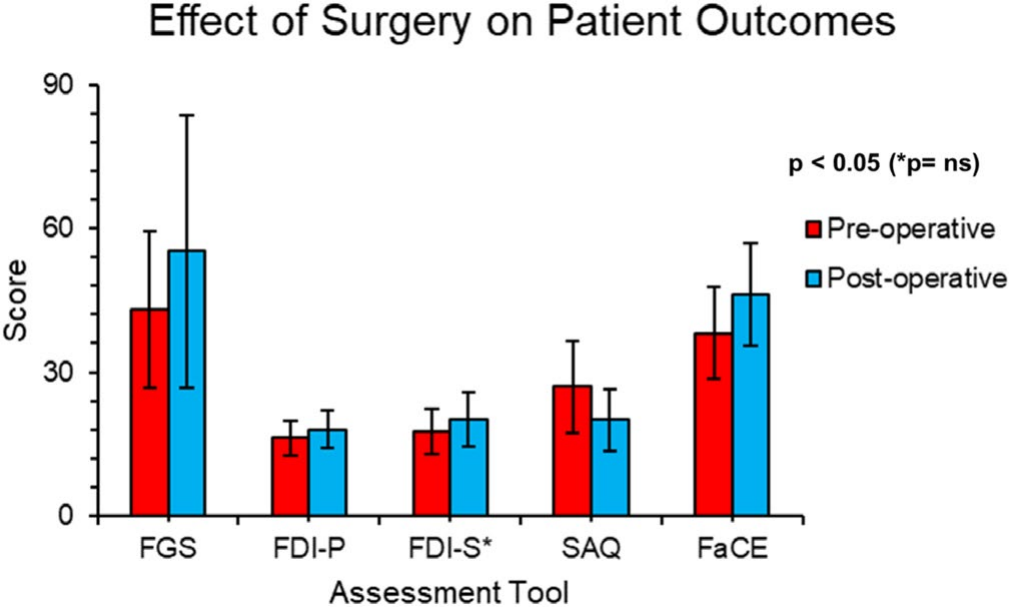
Bar chart of the effect of surgery on patient outcomes. Summarizes the overall improvement in the objective and subjective outcomes, following platysmal myoneurectomies ± selective neurectomies, with a *p* value from the student *t*‐test. (FaCE, facial clinimetric evaluation; FDI‐p, facial disability index physical; FDI‐s, facial disability index social; FGS, sunnybrook facial grading scale; SAQ, synkinesis assessment questionnaire).

**FIGURE 3:**
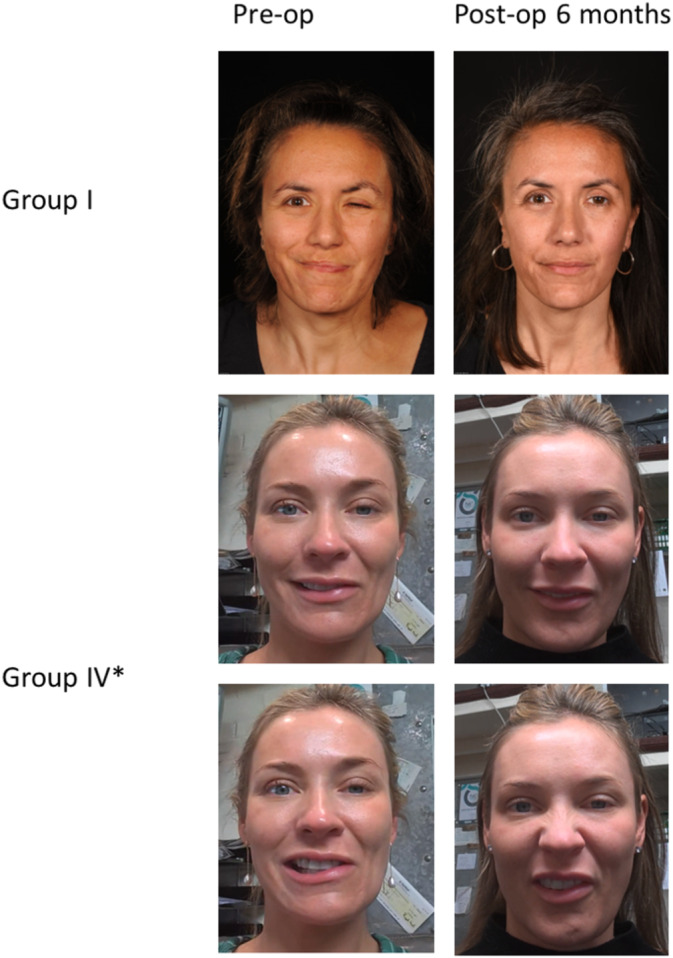
Pictures of a patient's physical outcomes pre‐operatively and 6 months post‐operatively after platysmal Myoneurectomy only (Group I) and platysmal Myoneurectomy + depressor anguli oris and buccinator selective neurectomy (Group IV). (* Group IV post‐operatively had botulinum toxin A (BTX‐A) injections).

Platysmal myoneurectomy improves objective (FGS) and subjective outcomes (FDI, SAQ, FaCE) (Figure 2). Figure 3 shows pictures of the physical improvement of a patient following a platysmal myoneurectomy (Group I) and platysmal myoneurectomy with DAO and buccinator selective neurectomy (Group IV). All results show a statistically significant improvement, apart from the FDI‐s improvement (*p* > 0.05). The FDI‐social score is a subjective data tool, and it reflects how patients feel and how they think society sees them (Van Swearingen and Brach 1996). This lack of statistical significance could indicate that patients view their condition qualitatively rather than quantitatively. Also, they may feel well in themselves but do not think people's views of them have changed, or vice versa, highlighting that the surgery reduces rather than cures symptoms.

**FIGURE 4:**
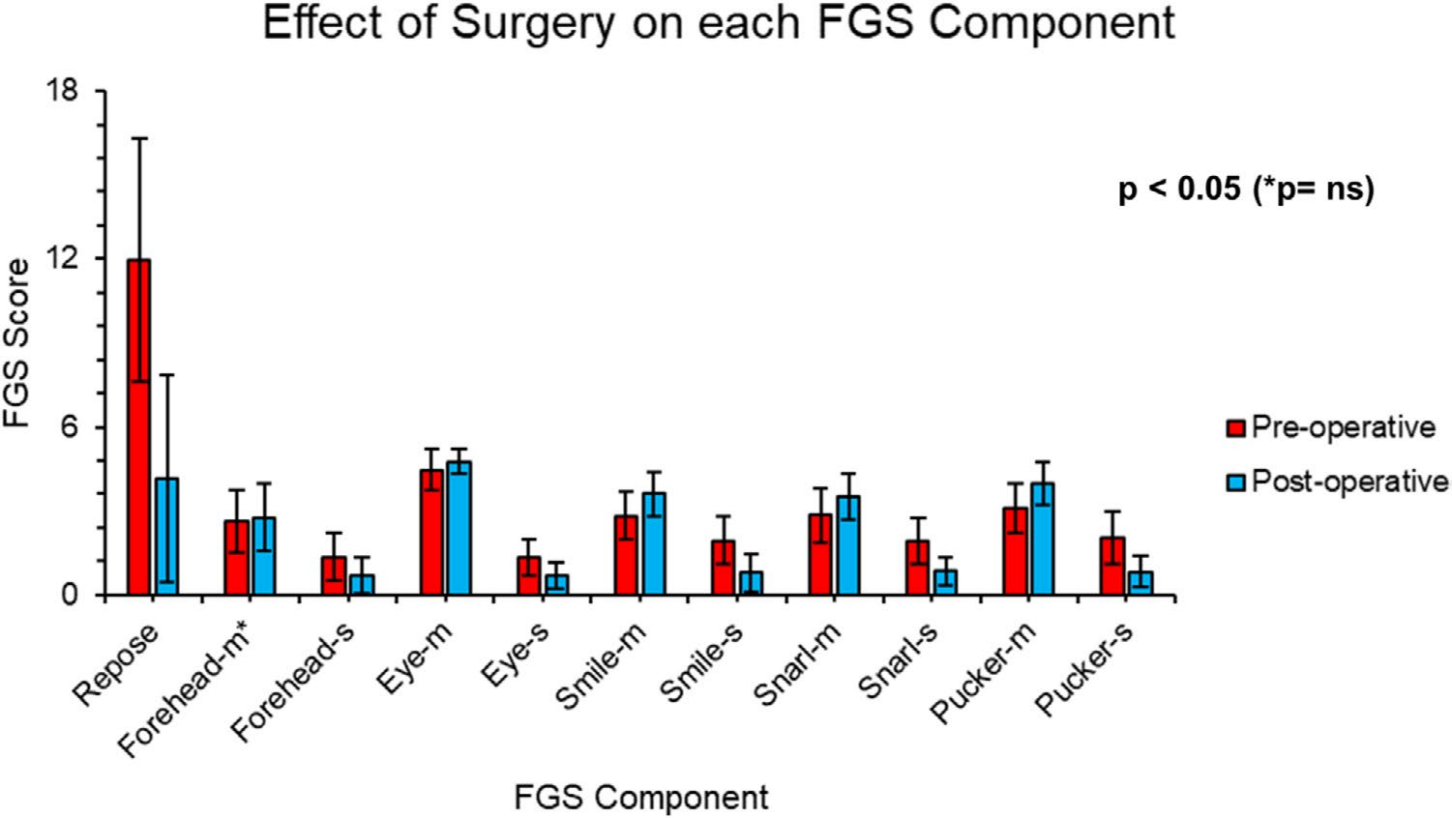
Bar chart of the effect of surgery on each FGS component. The site‐wise effect of platysmal myoneurectomies ± selective neurectomies with a *p* value from the student‐*t* test of each component. (FGS, sunnybrook facial grading scale; m, voluntary movement; ns, not significants; s, synkinesis).

This line of management showed a statistically significant improvement in all FGS components, apart from forehead movement (Figure 4). There is a statistically significant difference in voluntary movement in the lower two‐thirds of the face compared with the top one‐third (forehead and eye). However, synkinesis had a similar score in all assessed areas of the face, despite less improvement in forehead and eye synkinesis.

**FIGURE 5:**
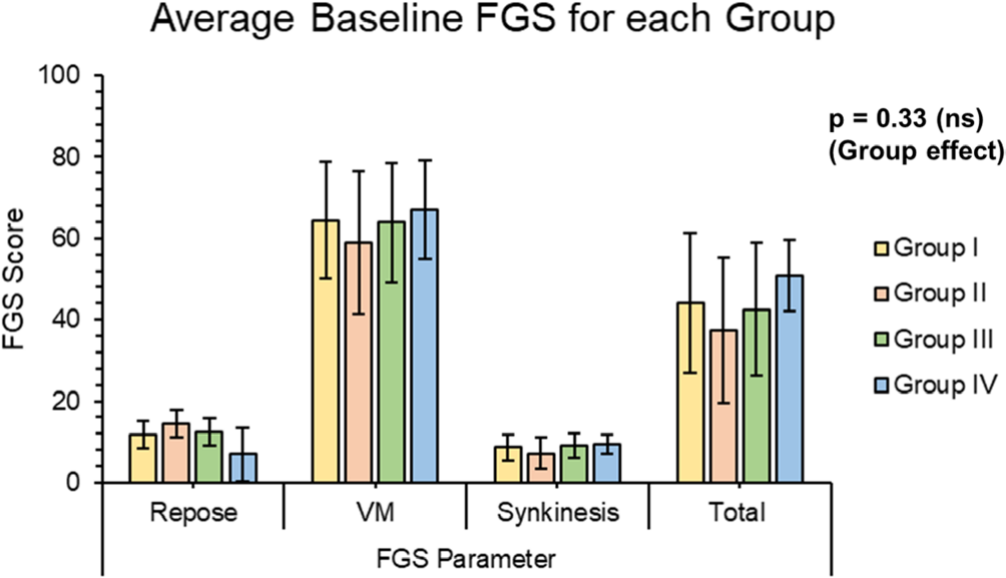
Bar chart of baseline FGS parameter outcomes for each surgical group. Baseline representation of the surgical groups, showing that their differences are not significant with *p* value from the two‐way ANOVA. (FGS, sunnybrook facial grading scale; ns, not significant; VM, voluntary movement).

A sub‐group FGS analysis of the surgical groups was performed starting with the pre‐op condition to match the groups. As shown in Figure 5, there was no statistical significance between Groups I and IV before treatment. Nevertheless, as there may be other confounders between groups, such as average age, year of surgery, and sex ratio between groups, which could risk random errors and potential confounding. The difference between baseline and post‐operative outcomes was calculated to reduce these errors and better assess how the surgery improves patient outcomes.

**FIGURE 6:**
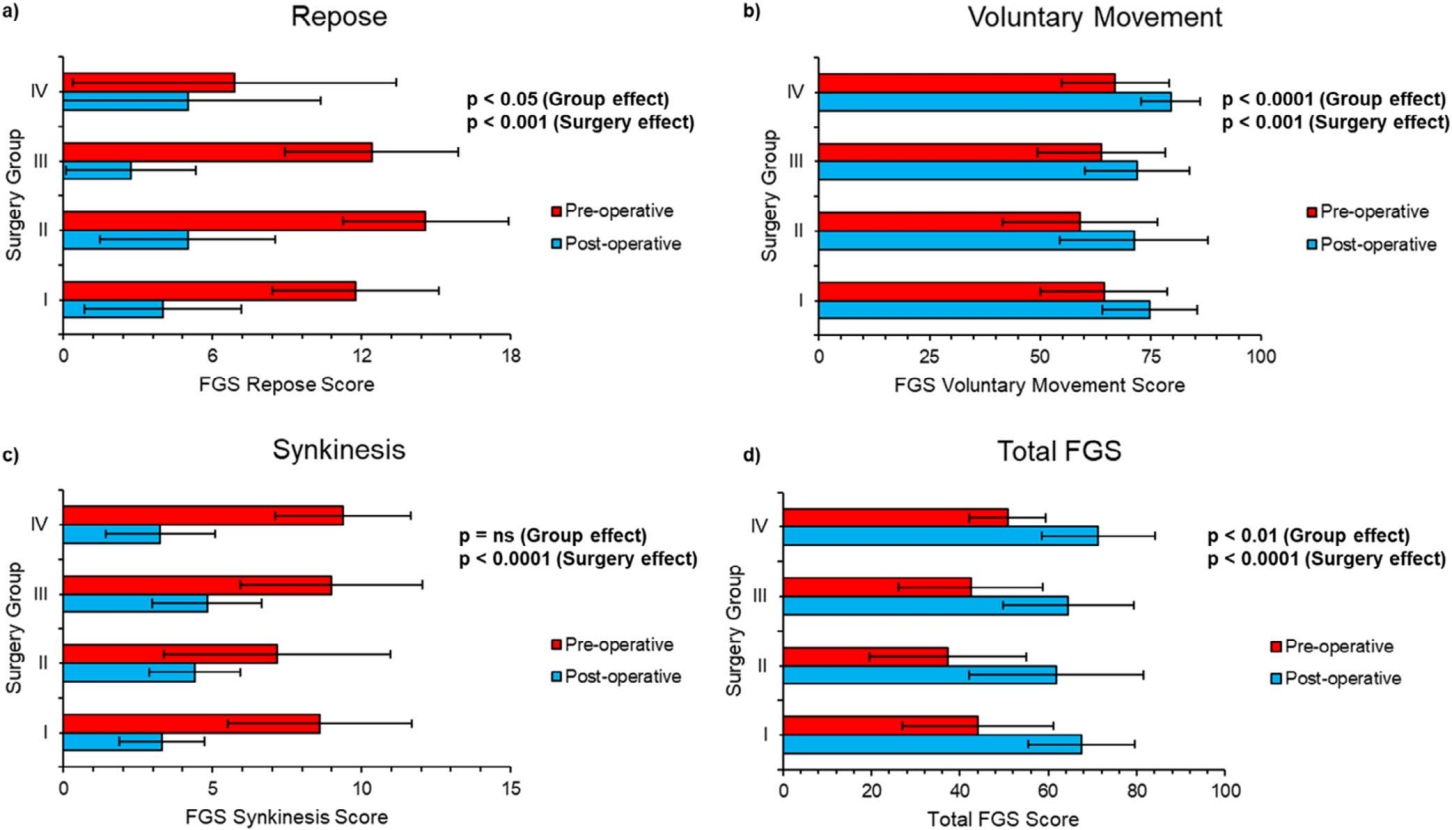
(a–d) Graphical representation pre‐ and post‐operative FGS parameter score in each surgical group. Shows improvement in FGS score with a two‐way ANOVA analysis of the group and surgery effect. (FGS, sunnybrook facial grading scale; ns, not significant).

All surgical variations significantly improve all FGS parameters (*p* < 0.05) (Figure 6). Furthermore, the group effect in repose, voluntary movement, and the total score are also statistically significant. Synkinesis does not have a statistically significant group effect, indicating that BTX‐A mapping was an effective allocation technique of surgical variations.

The neurectomy of the buccinator and DAO (Group IV) was the surgical variation that most significantly improved voluntary movement, although direct neurotization of the zygomaticus major muscle in Group II showed similar results, albeit with more variation. However, Group III had the best improvement in repose, while Group IV patients showed the greatest reduction in synkinesis. The release of the buccinator could be the determining factor here.

The overall improvement in the total FGS score showed no statistical difference across all sub‐groups, but in terms of maximal FGS values, sub‐group IV patients did best over time. A student *t*‐test was calculated between each surgical group's FGS parameter improvement to evaluate the statistical significance of each group's outcomes (Table 1). The selective neurectomy of the DAO and buccinator (Group IV) improves repose but significantly less than other sub‐groups in the initial post‐op stage. This, however, settles with time and becomes comparable with other sub‐groups.

**TABLE 1:** Table of the *p* values from the student *t*‐test for each FGS parameter between groups.

Groups	FGS parameter improvement student *t*‐test			
	Repose	VM	Synkinesis	Total
I versus II	ns	ns	ns	ns
I versus III	ns	ns	ns	ns
I versus IV	0.03	0.04	ns	ns
II versus III	ns	ns	ns	ns
II versus IV	ns	ns	0.02	ns
III versus IV	0.0007	ns	0.04	ns

Abbreviations: FGS, sunnybrook facial grading scale; ns, not significant; VM, voluntary movement.

Despite Figure 6 showing no significant group effect in synkinesis, the individual *t*‐tests (Table 1) comparing Group IV with Groups II and III were statistically significant (*p* < 0.05). The selective neurectomy of the DAO and buccinator (Group IV) showed greater improvement compared with the neurectomy of the DAO alone (Group III) and the ZM/LLS direct neurotization (Group II).

The patient‐related outcome measures (PROMs) show a statistically significant improvement in the surgical effects in all groups (Table 2). There was also a statistically significant improvement in the group effect across all PROMs apart from the FDI‐s. Each assessment tool has a different surgical group with the best outcome improvement, so there is no clear trend in which surgery patients perceive to be the most beneficial. The PROMs are subjective and assess different aspects of the patient's perception of their condition, which could explain this variation in results.

**TABLE 2:** Table of each surgical group's PROMs outcome pre‐ and postoperatively with a two‐way ANOVA for the group and surgery effect.

Score	Group	Pre‐operative		Post‐operative		Improvement	Two‐way ANOVA
		Mean	SD	Mean	SD		
FDI‐p	I	15.12	3.39	18.29	3.20	3.17	Group effect
	II	16.56	2.55	18.50	5.97	1.94	*p* = 0.002
	III	16.65	3.91	17.40	3.29	0.75	Surgery effect
	IV	17.50	3.46	22.5	2.12	5.00	*p* = 0.0006
FDI‐s	I	17.76	5.30	20.00	4.80	2.24	Group effect
	II	17.89	5.28	18.75	7.93	0.86	*p* = 0.11
	III	16.90	4.64	20.00	6.44	3.10	Surgery effect
	IV	18.87	4.19	23.50	0.71	4.63	*p* = 0.02
SAQ	I	26.10	9.63	21.17	8.64	4.93	Group effect
	II	26.75	11.61	17.75	6.13	9.00	*p* = 0.04
	III	26.46	9.91	19.00	5.07	7.46	Surgery effect
	IV	31.60	1.34	25.00	7.07	6.60	*p* = 0.006
FaCE	I	37.38	7.63	48.71	11.46	11.33	Group effect
	II	33.67	7.63	41.50	11.09	7.83	*p* = 0.003
	III	40.75	12.97	46.33	11.39	5.58	Surgery effect
	IV	38.57	5.91	40.00	7.07	1.43	*p* = 0.0001

Abbreviations: FaCE, facial clinimetric evaluation; FDI‐p, facial disability index physical; FDI‐s, facial disability index social; PROMs, patient related outcome measures; SAQ, synkinesis assessment questionnaire; SD, standard deviation.

From this cohort of sixty‐eight patients, there were no functional complications postoperatively. However, there were three hematomas (all of whom necessitated a return to theaters) and three superficial wound infections (managed conservatively), with a satisfactory outcome once managed.

## 4 Discussion

Selective neurectomy reduces facial nerve activity, resulting in decreased involuntary muscle contractions, which theoretically would reduce synkinesis (Shokri et al. 2021). In a study about selective neurectomy for refractory periocular synkinesis by van Veen et al., patients had reduced synkinesis and remained asymptomatic postoperatively (van Veen et al. 2018). Subsequently, Azizzadeh et al. performed a modified selective neurectomy that involved the distal branches of the buccal and cervical branches of the facial nerve (Azizzadeh et al. 2019). He later incorporated a platysmal myotomy and neurectomy of the buccal branch of the facial nerve into post‐facial palsy synkinesis management (Frants and Azizzadeh 2023). These surgeries produced long‐lasting results that improved smile and synkinesis without long‐term complications (Azizzadeh et al. 2019; Frants and Azizzadeh 2023). In these studies, patients had an asymptomatic period of about 1 year before requiring treatment such as chemodenervation (van Veen et al. 2018; Azizzadeh et al. 2019; Frants and Azizzadeh 2023).

In contrast to van Veen et al.'s and Azizzadeh et al.'s surgery, QVH incorporates facial therapy in the surgical process. In facial palsy, muscle fibers become chronically overactive, which causes actin and myosin cross‐bridges to remain intact, results in muscle stiffness, and may lead to stiffening; this process is called thixotropy (Lakie and Campbell 2019). The Three Element Hill model can explain the relationship between surgery and facial therapy because it represents what happens in a sustained muscle contraction (Battista et al. 2016). This model represents the relationship between a contractile element (actin and myosin cross‐bridges) and two non‐contractile elements (tendon and connective tissues) that together provide muscle elasticity (Battista et al. 2016). The platysmal myoneurectomy only targets the non‐contractile element, but facial therapy addresses the contractile element by reducing thixotropy (Lakie and Campbell 2019; Battista et al. 2016). Furthermore, the significantly beneficial role of facial therapy in the postoperative rehabilitation period of selective neurectomy patients is well documented, contributing to an overall improvement in function and pain (Neville et al. 2022).

Platysmal myoneurectomy does not cure synkinesis but releases tension between muscles and nerves that reduces it. This tension release improves voluntary movement and repose, causing an improvement in the smile, function, and facial symmetry. Theoretically, if these were improved, surgery would then improve mental health status in patients post‐operatively by targeting these distressing side effects of facial palsy (Pasquale et al. 2021). In this study, social outcomes improved within each surgical group, but when compared with the overall data, there was no statistically significant improvement.

The DAO and buccinator selective neurectomy with the platysmal myoneurectomy (Group IV) had superior outcome improvement in voluntary movement and synkinesis compared with the other surgical groups. The buccinator is the main anatomical difference between Group IV and the other groups. Group IV surgery outcomes may be influenced by the “ping effect,” potentially resulting in confounding (Simon et al. 2020). This effect refers to the release of the platysma, DAO, and buccinator causing the opposing facial muscles (ZM and LLS) to contract too much and pull up the cheek as a compensatory mechanism (Simon et al. 2020). This effect is temporary and starts at ~3 months and is usually controllable by 9 months post‐operatively. Since the initial data from this study was from the 3‐month appointments, the patient's presentation in Group IV could have been affected by the “ping effect” that contributed to their outcomes. To investigate the “ping effect,” the data from Group IV was assessed over a period of around 22 months, and a greater improvement in synkinesis was found. Therefore, this means the “ping effect” does happen but settles with time and consistent facial rehabilitation.

The previously mentioned studies of van Veen et al. and Azizzadeh et al. measured longer‐term impacts like the asymptomatic period and BTX‐A doses post‐operatively (van Veen et al. 2018; Azizzadeh et al. 2019; Frants and Azizzadeh 2023). Assessing this for platysmal myoneurectomies would help determine how it impacts patients long term. An FGS score could be calculated at every facial therapy appointment to monitor facial function progressively and record when/if patients require chemodenervation post‐operatively. Furthermore, the FDI‐s in this study raised some questions about the impact of the surgical process on mental health. Mental health issues are significant in facial palsy patients, so an in‐depth assessment of a patient's mental health would allow greater insight into this (Pasquale et al. 2021). Ideally, this would be with tailored questionnaires for post‐facial palsy synkinesis and specialist psychological evaluations to provide objective and subjective outcomes.

## 5 Conclusion

Platysmal myoneurectomy is the baseline operation that improves symmetry, voluntary movement, and synkinesis. Preliminary data on Group IV suggest that the neurectomy of the DAO and buccinator with the resection of the platysma is the best option to improve synkinesis. However, this method causes a reactionary smile‐mimetic muscle tightening: the “ping effect,” which resolves with time. There are no functional complications secondary to this surgery.

## Conflicts of Interest

Charles Nduka: Founder and Chief Scientific Officer, Emteq Labs, UK. Co‐founder and CEO, Facial Palsy UK charity (unsalaried). Co‐editor of the book, The Management of post‐facial paralysis synkinesis, 2021, published by Elsevier (royalties donated to FPUK). Grants: Various grants ~$1.5 M in the last 3 years from Innovate UK, National Institute of Health Research (NIHR), and Horizon 2020 for technology development (none relating to the subject matter). Patents: 23 patents, none relating to the subject matter. Maria Paranhos Barbot, Catriona Neville, Tamsin Gwynn, Karen Young, Crystal Selley‐West, Raman Malhotra, Ruben Yap Kannan: Declare no conflicts of interest.

## Data Availability

The data that support the findings of this study are available on request from the corresponding author. The data are not publicly available due to privacy or ethical restrictions.
